# Changes in the gut microbiota mediate the differential regulatory effects of two glucose oxidases produced by *Aspergillus niger* and *Penicillium amagasakiense* on the meat quality and growth performance of broilers

**DOI:** 10.1186/s40104-020-00480-z

**Published:** 2020-07-06

**Authors:** Shengru Wu, Xiaodong Chen, Taohuan Li, Hao Ren, Lixin Zheng, Xiaojun Yang

**Affiliations:** 1 College of Animal Science and Technology, Northwest A&F University, Yangling, Shaanxi China; 2 Center for Translational Microbiome Research, Department of Molecular, Tumor and Cell Biology, Karolinska Institutet, Stockholm, Sweden; 3 Jiangxi Zhengbang Technology Co., Ltd., Nanchang, Jiangxi China

**Keywords:** Broiler, Glucose oxidase, Growth performance, Gut function, Gut microbiota

## Abstract

**Background:**

Glucose oxidase (GOD), an aerobic dehydrogenase, has been used as an antibiotic substitute in feed. A study was conducted to evaluate the differential effects of 2 different GODs fermented by *Aspergillus niger* or *Penicillium amagasakiense* on caecal microbiota and to further illuminate the potential roles of changes in the gut microbiota in regulating the growth performance and meat quality of broiler chickens.

**Results:**

A total of 420 one-day-old healthy Arbor Acres broilers were randomly assigned to 4 treatments: the control group, the antibiotic growth promoter (AGP) supplementation group, and the GOD-A and GOD-P (GODs produced by *A. niger* and *P. amagasakiense*, respectively) groups. As a result, supplementation with GOD produced by *P. amagasakiense* could significantly improve the average daily weight gain and average daily feed intake of broilers before 21 days of age by significantly increasing the enzymatic activities of jejunal amylase and those of ileal amylase, chymotrypsin, and lipase in 21-day-old broilers and could increase the enzymatic activities of duodenal amylase, jejunal amylase and lipase, and ileal chymotrypsin and lipase in 42-day-old broilers. Meanwhile, compared with AGP treatment, supplementation with GOD produced by *P. amagasakiense* significantly decreased the L value of 21-day-old broilers and the ΔpH and L* value of 42-day-old broilers, while supplementation with GOD produced by *A. niger* significantly increased the pH_24 h_ value of 21-day-old and 42-day-old broilers by reducing plasma malondialdehyde content. By using 16S rRNA sequencing, we found that the beneficial bacteria and microbiota in broilers were not disturbed but were improved by GOD supplementation compared with ADP treatment, including the genera *Eubacterium* and *Christensenella* and the species *uncultured_Eubacterium_sp*, *Clostridium_asparagiforme*, and *uncultured_Christensenella_sp*, which were positively related to the improved intestinal digestive enzymatic activities, growth performance, and meat quality of broilers.

**Conclusion:**

The altered gut microbiota induced by supplementation with glucose oxidase produced by *P. amagasakiense* mediate better regulatory effects on the meat quality and growth performance of broilers than that induced by supplementation with glucose oxidase produced by *A. niger*.

## Introduction

The ban on antibiotic growth promoters (AGPs) has prompted research into developing alternative effective additives aimed at promoting growth performance and maintaining the intestinal homeostasis of broilers, including immune, microbial, and functional homeostasis [1, 2]. Glucose oxidase (GOD), an aerobic dehydrogenase that can specifically oxidize β-*D*-glucose into gluconic acid, produce hydrogen peroxide and then consume large amounts of oxygen, could be beneficial to maintain intestinal microbiome balance and to defend against conditioned pathogen proliferation [3]. The intestinal digestive and absorption functions and growth performance of animals could also be improved by using GOD as an alternative to AGPs according to our previous study [4]. Furthermore, over the past decades, the production of poultry meat has been rapidly increasing worldwide. However, today’s consumers are increasingly concerned about the quality and safety of meat. The GODs consume large amounts of oxygen in the gut and could therefore serve as important antioxidants in animals by eliminating oxygen free radicals [3, 4]. Hence, the effects of additives successfully used as alternatives to AGPs, mainly the alteration of the antioxidant capacity and the improvement of meat quality of broilers, should also be further considered [5, 6].

The gastrointestinal tract is colonized by diverse microbiota, which have increasingly been reported to be associated with “intestinal” or “non-intestinal” functions, such as processes ranging from absorption to metabolism, immune activation, and even gut-brain axis function [7]. In the gut, microbial homeostasis could defend against conditioned pathogen proliferation and invasion, stimulating and enhancing intestinal digestion-absorption functions and activating the immune response [7, 8]. The gut microbiota is easily influenced or regulated by environmental changes, especially dietary changes and supplementation with forbidden AGPs [9, 10]. Specifically, as a potential AGP substitute in feed, the beneficial roles of GOD could be inseparable from the important roles of intestinal microbiota.

GOD can be fermented by *Aspergillus niger* and *Penicillium amagasakiense*, resulting in quite different structures and enzyme kinetics [11]. In detail, the molecular weights of the GODs produced by *A. niger* and *P. amagasakiense* were 152 and 150 kDa, respectively. *A. niger* GOD contained more mannose and hexosamine than *P. amagasakiense* GOD but less glucose. The overall carbohydrate content was found to be 16% for *A. niger* GOD and 11% for *P. amagasakiense* GOD. An analysis of the amino acid content of the two enzymes revealed that *A. niger* GOD contained more histidine, arginine and tyrosine and less lysine and phenylalanine than *P. amagasakiense* GOD [11]. The optimum pH ranges for GOD from *A. niger* and *P. amagasakiense* were shown to be 3.5–6.5 and 4.0–5.5, respectively. It is evident that GOD from *A. niger* has a broader pH range than that from *P. amagasakiense* GOD [11–13]. These differential characteristics may determine their different roles in the gut, including altering gut microbiota and improving growth performance and meat quality. Therefore, the present study was carried out to identify the differential effects of GOD produced by *A. niger* and *P. amagasakiense* on the growth performance, meat quality, and gut microbiota of broiler chickens, which could provide additional information on the use of GOD as an AGP substitute in broiler feed, especially the differential functions of GOD produced by *A. niger* and *P. amagasakiense*.

## Materials and methods

### Birds and experimental design

The bird maintenance and experimental protocols in this study were approved by the Institutional Animal Care and Use Committee of Northwest A&F University (protocol number NWAFAC1378). GODs were fermented by *P. amagasakiense* or *A. niger* and then purified and were provided by Xia Sheng Industrial Group Co., Ltd. (Ningxia, China).

Based on a single factor experimental design, a total of 420 1-day-old Arbor Acres broiler chicks were randomly assigned to 4 groups with seven replications per treatment and 15 birds per replicate, including the control group (CON), antibiotic growth promoter (AGP) supplementation group, and 2 GOD supplementation groups. Briefly, the CON group was provided a corn and soybean basal diet, the AGP group was provided antibiotic growth promoters (20 mg colistin sulfate, 6 mg enamycin and 40 mg salicin sodium premix per kilogram diet) plus the basal diet, and the GOD-A and GOD-P groups were provided 2 different kinds of GOD produced by *A. niger P. amagasakiense* (60 U GOD per kilogram diet) plus the basal diet [4]. The GOD enzyme unit was measured by using the Micro Glucose oxidase (GOD) Assay Kit (Solarbio, Beijing, China). Additionally, the differences between the 2 different types of GOD were in accordance with a previous report [11]. The basal starter ration (0 to 3 weeks) and finisher ration (4 to 6 weeks) were both typical of mash diets commonly used in the Northwestern District of China that met the National Research Council (NRC, 1994) recommendations. The ingredient and chemical composition of the basal diets are also presented in Table 1. All chickens were kept in an environmentally controlled henhouse with double-floor metabolism cages, and the water and feed were available ad libitum. For the first week, the animals were exposed to a 24-h photoperiod, and then they were exposed to an intermittent photoperiod with 1 h of light and 2 h of darkness at 2–6 weeks. The brooding temperature was maintained at 36 °C for the first week, then reduced by 4 °C each week and maintained at 24 °C from 4 to 6 weeks.

**Table 1:** Composition and nutrient levels in the basal diet (air-dry basis)

Ingredients, %	1 to 3 weeks	4 to 6 weeks	Nutrient Levels, %^2^	1 to 3 weeks	4 to 6 weeks
Corn	56.00	53.50	ME, MJ/kg	12.14	12.77
Soybean meal (contains about 43% crude protein)	26.60	22.00	Crude protein (CP)	21.25	19.30
Flour	6.00	8.00	Calcium (Ca)	0.90	1.16
Corn gluten meal	4.20	1.70	Total phosphorus (TP)	0.65	0.62
DDGS-28	3.00	5.00	Available phosphorus (AP)	0.43	0.35
CaHPO_4_	1.65	0.28	Ether extract (EE)	2.61	6.39
CaCO_3_	1.08	1.01	sodium chloride (NaCl)	0.26	0.29
*L*-Lysine H_2_SO_4_	0.33	0.24	Lysine	1.24	1.08
Premix^1^	0.45	0.45	Methionine	0.52	0.50
Salt	0.20	0.20	Methionine and cystine	0.86	0.79
*DL*-Methionine	0.19	0.21	Threonine	0.87	0.70
*L*-Threonine	0.10	0.11			
NaHCO_3_	0.10	0.10			
Choline chloride	0.08	0.08			
Phytase	0.02	0.02			
Mixed oil	–	2.60			
Meat and bone meal	–	2.00			
Instant noodle	–	2.00			
Feather meal	–	0.50			
Total	100.00	100.00			

^1^The premix provided the following nutrients per kg diet: vitamin A, 11,600 IU; vitamin D_3_, 3,360 IU; vitamin K_3_, 4.02 mg; vitamin B_1_, 2.2 mg; vitamin B_2_, 7.3 mg; vitamin B_6_, 4.9 mg; vitamin B_12_, 0.014 mg; Cu, 8.4 mg; Zn, 49.5 mg; Fe, 54.0 mg; Mn, 150.0 mg

^2^The contents of CP, Ca, TP and EE were measured by the feed proximity analysis method, and the contents of other nutrients were calculated

### Growth performance

The experiment lasted for 42 days. On day 21 and day 42, chickens were weighed by pen (replication), and the feed consumption of each replication was recorded. Average daily weight gain (ADG), average daily feed intake (ADFI), and ratio of feed to gain (F/G; FCR) were calculated for the periods from day 1 to day 21, from day 22 to day 42, and from day 1 to day 42.

### Sample collection

We collected samples on days 21 and 42, and the birds were humanly fasted for 12 h before sampling. For each sampling, two birds from each selected replicate were randomly selected. Blood samples from these broilers were collected, and plasma samples were prepared. Then, these birds were killed and rapidly dissected. Two birds from each replicate were used to collect different samples. One broiler from each replication was sacrificed by exsanguination after intravenous administration of 3% sodium pentobarbital (25 mg/kg body weight; Sigma, USA) and immediately dissected to collect duodenal, jejunal, and ileal content samples, and another broiler was sacrificed and immediately dissected to collect duodenal, jejunal, and ileal mucosa samples. Moreover, the caecal contents, whole left breast muscle, and left drumstick muscle from both broilers from each replicate were collected. Intestinal samples were collected into 2 mL Eppendorf tubes, frozen immediately in liquid nitrogen and then stored at − 80 °C until analysis.

### Measurement of meat quality of breast and drumstick muscles from 42-day-old broilers

The collected breast and left drumstick muscles were immediately used to measure meat quality. Muscle pH was analysed using a portable pH metre (Testo 205, Germany) at 45 min (initial pH; pH_45 min_) and 24 h postmortem (ultimate pH; pH_24 h_). The pH metre was calibrated using the 2-point method against standard buffer solutions with pH values of 4.0 and 7.0. The pH value was expressed as the average of the 3 measurements. At 24 h postmortem, meat colour was measured by the Chroma metre CR-410 (Minolta, Suita-shi, Japan). Colour was measured at 3 locations on the dorsal side of the breast fillet and at 3 locations on the medial (bone) side of thigh. Colour was reported as L*, a*, and b* values [14].

The water-holding capacity of meat was estimated by measuring the drip loss of raw meat after storage: the pectoralis major muscle was weighed 24 h postmortem and immediately placed in a plastic bag, hung from a hook, and stored at 2 °C for 4 days. After hanging, the sample was wiped with absorbent paper and weighed again. The difference in weight corresponded to the drip loss and was expressed as the percentage of the initial muscle weight.

The muscles were stored at 4 °C until 24 h postmortem, and then these samples were used to measure shear force. The shear force value was measured as described by Chen et al. [15] using a digital meat tenderness metre (model C-LM3, Northeast Agricultural University, Harbin, China). Each sample was measured 3 times, and the average of 3 readings was used as the shear force value of the sample and was expressed in Newtons.

### Determination of antioxidant activities in plasma

The activities of glutathione peroxidase (GSH-Px) and superoxide dismutase (SOD), the total antioxidant capacity (T-AOC), and the malonaldehyde (MDA) concentration of plasma were measured by a spectrophotometric method according to methods previously described in detail [16].

### Measurement of the activities of small intestinal digestive enzymes

The sample preparation and the activities of small intestinal digestive enzymes, including amylase, chymotrypsin, and lipase of the duodenum, jejunum, and ileum, were measured by spectrophotometric methods according to the manufacturer’s procedure (Jiancheng Biological Engineering Research Institute, Nanjing, China).

### DNA extraction

Five of seven replications were randomly selected, and fifty caecal content samples from 21-day-old broilers and fifty caecal content samples from 42-day-old broilers were used for DNA extraction using the QIAmpH DNA Stool Mini Kit (Qiagen, Germany) according to the manufacturer’s instructions. A Nanodrop ND-1000 spectrophotometer (Thermo Scientific, USA) was used to assess the DNA quantity of the DNA samples. According to the DNA concentration, equal quantities of two DNA samples extracted from two caecal content samples from the same replicate were pooled into one DNA sample for further sequencing. The quantity and quality of the 40 pooled DNA samples (20 samples from 21-day-old broilers and 20 samples from 42-day-old broilers) was further assessed by a Nanodrop ND-1000 spectrophotometer (Thermo Scientific, USA), and then, the samples were stored at − 80 °C until sequence analysis.

### 16S rRNA amplification of V3 + V4 region and Illumina sequencing

16S rRNA amplification and sequencing was performed as described in a previous report [17]. Briefly, the V3 + V4 hypervariable region of the 16S rRNA gene was PCR amplified from microbial genomic DNA harvested from caecal content samples using the forward primer 349 F (5′-CCTAYGGGRBGCASCAG-3′) and the reverse primer 806 R (5′-GGACTACNNGGGTATCTAAT-3′). Barcodes, an eight-base sequence unique to each sample that was designed by Genedenovo Inc. (Guangzhou, China), were added to each primer for sample identification. PCR amplifications were carried out using TransStart Fastpfu DNA Polymerase (Qiagen, China) and amplified in a 20-μL reaction containing 4 μL 5× FastPfu Buffer (containing 1.8 mmol/L MgCl_2_), 4 μL dNTPs (2.5 mmol/L), 0.8 μL forward primer (5 μmol/L), 0.8 μL reverse primer (5 μmol/L), 0.4 μL FastPfu Polymerase and 10 ng DNA template. The PCR included an initial denaturation step at 95 °C for 5 min followed by 27 cycles of 95 °C for 30 s, 55 °C for 30 s and 72 °C for 30 s and a final extension step at 72 °C for 10 min in a GeneAmp® 9700 (ABI, USA). The PCR product (approximately 400 bp) was excised from a 2% agarose gel stained with ethidium bromide, and purification was performed using the QIAquick Gel Extraction Kit (Qiagen, USA). DNA quality and concentration were measured using a Quantus Fluorometer (Promega, USA) and then quantified using a Quant-iT PicoGreen dsDNA Assay kit (Thermo, USA) according to the manufacturer’s instructions. Purified amplicons were pooled according to data size and the quantified results of each sample. Finally, paired-end sequencing was conducted on an Illumina HiSeq 2500 PE250 (Illumina, USA) according to the standard protocols by Genedenovo Inc. (Guangzhou, China).

### Illumina sequencing data processing

After sequencing, we separated the data for all of the samples according to the barcode sequence. Then, all barcodes and adapters were removed, and all reads were quality assessed. The raw data obtained after sequencing included dirty reads containing adapters or low-quality bases, which would affect the subsequent assembly and analysis. Thus, to obtain high-quality clean reads, raw reads were further filtered according to the following criteria: 1) reads containing more than 10% of unknown nucleotides (N) were removed; and 2) reads containing less than 80% of bases with quality (Q-value)>20 were removed. The filtered reads were then assembled into tags according to overlap between paired-end reads with an overlap of more than 10 bp and a mismatch of less than 2%. Mothur (v.1.34.0) software [18] was used to remove redundant tags to obtain unique tags. The obtained unique tags were then used to calculate the abundance. Rdp Classifier (http://rdp.cme.msu.edu/classifier/classifier.jsp) was used to classify tags into different taxonomies against the GreenGenes Database (version 20101006) with a confidence threshold of 0.5 [19]. The operational taxonomic unit (OTU)-based method, in which sequences are split into bins based on taxonomy and clustered to each bin with a cutoff of 0.03 [20], was used for analysis. The taxonomic classification of OTUs was based on the annotation of tags according to the mode principle, that is, the taxonomic rank that contained more than 66% of tags was thought to be the taxonomic rank of this OTU, and otherwise the higher rank would be considered. The rarefaction curve was used to evaluate whether the sequencing data amount was enough to cover all of the sample species and to reflect the species richness in samples. Alpha diversity analysis consisting of community diversity (Inverse Simpson and Shannon) and richness (OTU number observed, Chao 1 and ACE) was performed using Mothur based on a summary single command [18, 21]. The microbiota were compared for beta diversity using the distance matrices generated from weighted UniFrac analysis, principal coordinated analysis (PCoA) and ANOMIS analysis. The Mann-Whitney U test (*P* < 0.05) was used to identify differences between the samples.

### Statistical analysis

The analysis was done by using one-way ANOVA with SPSS 21.0 software with replicates as experimental units, and differences were considered to be statistically significant at *P* < 0.05. Significant differences at the 0.05 level due to treatments were identified by Duncan’s multiple range tests.

### Data availability

The sequence data were deposited and are available in the Sequence Read Archive (SRA) of NCBI under the accession project number PRJNA606870.

## Results

### GOD produced by *P. amagasakiense* could significantly improve the growth performance of broilers

**Fig. 1:**
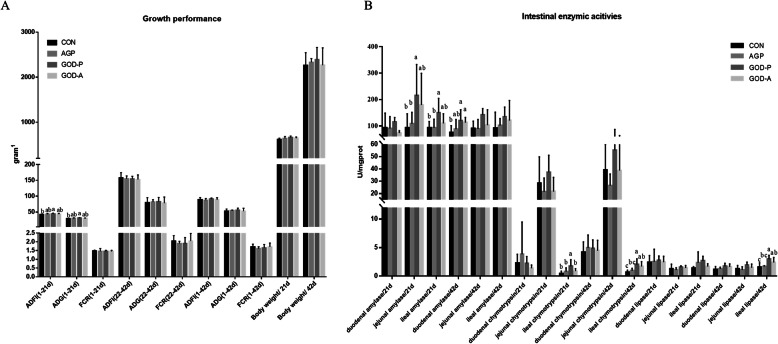
Effects of two different types of GODs and AGPs on the growth performance (**a**) and intestinal enzyme activities (**b**) of 21-day-old and 42-day-old broilers. Note: CON indicates the control group, AGP represents the data from broilers fed antibiotic growth promoters, and GOD-P and GOD-A represent the data from broilers fed GOD produced by *Aspergillus niger* and *Penicillium amagasakiense* respectively. ADG: average daily weight gain; ADFI: average daily feed intake; FCR: ratio of feed to gain; ^1^ except for FCR, which has no unit, the unit of other indices in (**a**) are all expressed in grams

Compared with the control diet, supplementation with AGPs and GOD produced by *A. niger* did not significantly increase the growth performance of broilers during the experimental period of 6 weeks. However, compared with the control diet, supplementation with GOD produced by *P. amagasakiense* significantly improved the ADG and ADFI of broilers before 21 days of age (Fig. 1A). Moreover, there were no significant differences identified among the AGP and 2 different GOD supplementation groups.

Furthermore, changes in small intestinal digestive enzymatic activities were further analysed (Fig. 1B). Compared with the control diet, supplementation with AGPs had no significant effects on intestinal digestive enzymatic activities. Meanwhile, the group supplemented with GOD from *A. niger* showed significantly increased duodenal amylase and ileal chymotrypsin and lipase activities compared with the CON group at 42 days. Moreover, compared with the CON diet, supplementation with GOD produced by *P. amagasakiense* significantly increased the enzymatic activities of jejunal amylase and of ileal amylase and chymotrypsin in 21-day-old broilers. Supplementation with GOD produced by *P. amagasakiense* could increase the enzymatic activities of duodenal amylase and of ileal chymotrypsin and lipase in 42-day-old broilers. Similarly, compared with AGP supplementation, supplementation with GOD from *P. amagasakiense* could significantly increase jejunal and ileal amylase and chymotrypsin activities in 21-day-old broilers and increase ileal lipase and chymotrypsin activities in 42-day-old broilers. Compared with supplementation with GOD from *A. niger*, supplementation with GOD from *P. amagasakiense* significantly increased ideal chymotrypsin in 21-day-old broilers.

### Different types of GODs produced by *A. niger* and *P. amagasakiense* have differential regulatory roles in broiler meat quality

**Fig. 2:**
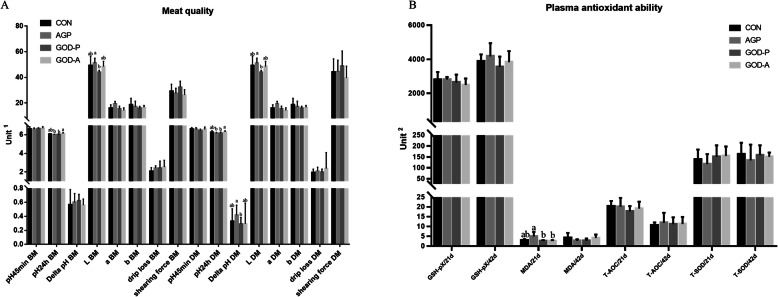
Effects of two different types of GODs and AGPs on the meat quality of breast muscle (BM) and drumstick muscle (DM) (**a**) and on the plasma antioxidant activity (**b**) of 21-day-old and 42-day-old broilers. Note: CON indicates the control group, AGP represents the data from broilers fed antibiotic growth promoters, and GOD-P and GOD-A represent the data from broilers fed GOD produced by *Aspergillus niger* and *Penicillium amagasakiense*, respectively. GSH-Px: glutathione peroxidase, SOD: superoxide dismutase, T-AOC: total antioxidant capacity, MDA: malonaldehyde. ^1^The units in (**a**) are as follows: drip loss (%) and shear force (N)*.*^2^ The units for each index are as follows: T-AOC (U/mL); GSH-Px (μmol/mL); SOD (U/mL); and MDA (nmol/mL)

The different roles of the two different types of GODs produced by *A. niger* and *P. amagasakiense* in regulating the breast and drumstick meat quality of 42-day-old broilers were evaluated (Fig. 2A). Compared with the control group, these supplementation groups showed no significant changes in meat quality indices in the present study. However, compared with the AGP groups, the groups supplemented with GOD produced by *P. amagasakiense* showed significantly decreased L value of breast muscle and ∆pH and L value of drumstick muscle, while the group supplemented with GOD produced by *A. niger* showed significantly increased pH_24 h_ values of breast and drumstick muscle. Meanwhile, significantly decreased pH_24 h_ values of the breast and drumstick were identified in broilers supplemented with GOD produced by *P. amagasakiense* compared with those supplemented with GOD produced by *A. niger* (Fig. 2A)*.*

Furthermore, considering that GOD could influence the intestinal environment by utilizing oxygen and producing gluconic acid and hydrogen peroxide, we further measured plasmatic indices related to antioxidant ability (Fig. 2B), which could also contribute to regulating meat quality. In the present study, the MDA content of 21-day-old broilers was significantly decreased in the GOD groups (produced by *A. niger* and *P. amagasakiense*) compared with the AGP group, while T-AOC and the enzymatic activities of T-SOD, and GSH-Px, were not changed in the present study.

### Different types of GODs could significantly alter the gut microbiota

**Fig. 3:**
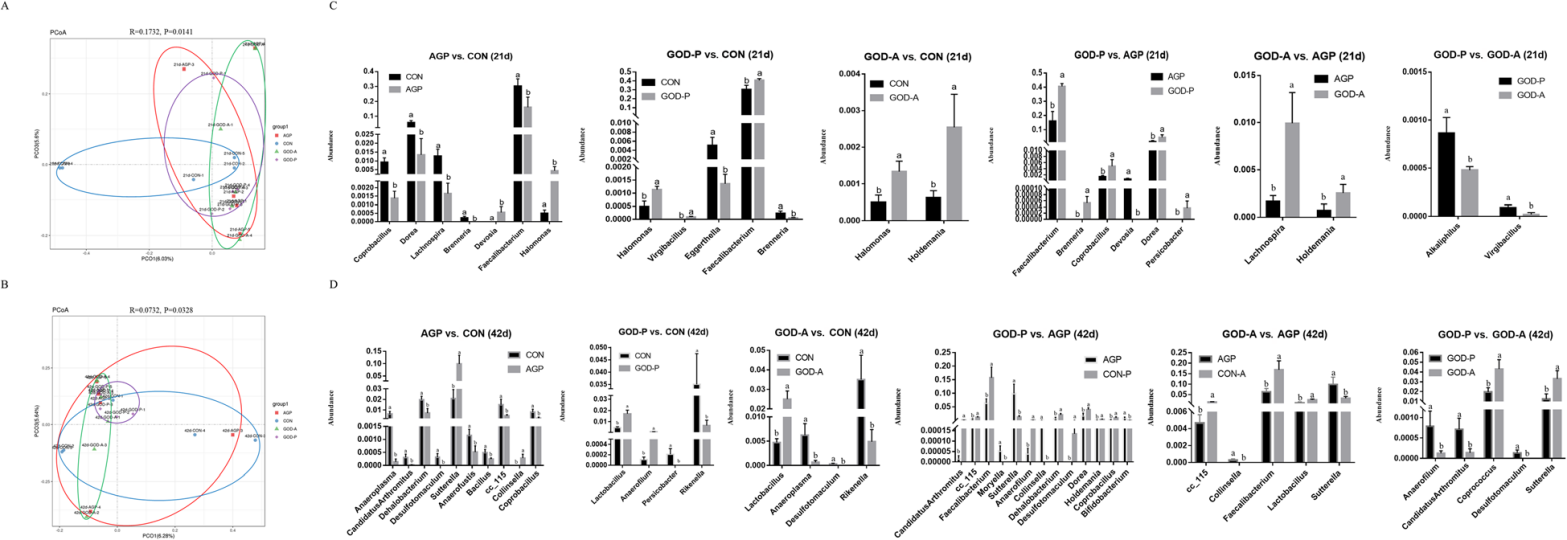
Beta diversity analysis (**a** and **b**) based on principal coordinate analysis (PCoA) analysis and the differential genera identified between groups using the Mann-Whitney U test (**c** and **d**). Note: CON indicates the control group, AGP represents the data from broilers fed antibiotic growth promoters, and GOD-P and GOD-A represent the data from broilers fed GOD produced by *Aspergillus niger* and *Penicillium amagasakiense*, respectively

By using 16S rRNA sequencing, the role of the two types of GODs in regulating gut microbiota was evaluated. According to the α-diversity results, we found that supplementation with GOD produced by *A. niger* significantly increased the abundance of the caecal microbiota (Chao and ACE indices) of 21-day-old broilers compared with the other 3 treatments, and supplementation with AGP decreased the abundance (Chao and ACE indices) and diversity (Shannon index) of the caecal microbiota of 21-day-old broilers compared with the CON and GOD-A diets (Table 2). However, the abundance and diversity of caecal microbiota in 42-day-old broilers were not changed in the present study (Table 2). Furthermore, a significant distinction among the CON, AGP, and 2 GOD groups was identified (*P* = 0.0141) by principal component analysis, which implied that the intestinal microbiota was significantly altered by GOD and AGP supplementation (Fig. 3A and B). The AGP and two GOD groups were clustered and separate from the CON group at 21 and 42 days, which indicated that the antibiotics and GOD have selective effects on the microbiota (Fig. 3A, B). Meanwhile, all 5 samples from the 42-day-old broilers supplemented with GOD produced by *P. amagasakiense* clustered better than those from the CON and GOD-A groups (Fig. 3B). Overall, both antibiotics and GOD could influence the gut microbiota, and the different roles of antibiotics and two different GODs in regulating microbiota warrant further study.

**Table 2:** Effects of two different types of GODs and AGPs on the alpha diversity indices of caecal microbiota

Item^1^	CON	AGP	GOD-P	GOD-A	SEM^2^	*P-*value
21-day-old broilers						
Chao	2968.3^ab^	2415.3^b^	2915.4^ab^	3538.8^a^	135.27	0.018
Ace	4045.0^b^	3209.4^c^	4048.3^b^	5110.8^a^	214.39	0.007
Shannon	5.01^a^	4.37^b^	4.76^ab^	5.05^a^	0.10	0.045
Simpson	0.023	0.060	0.033	0.026	0.007	0.192
42-day-old broilers						
Chao	4320.1	4441.3	3112.4	3371.8	330.18	0.407
Ace	6034.1	6500.4	3973.4	4402.1	593.84	0.384
Shannon	4.98	4.74	4.98	4.97	0.09	0.793
Simpson	0.041	0.042	0.033	0.036	0.005	0.929

^a-b^Mean values within a row with the same superscript letters were not significantly different (*P* < 0.05)

^1^AGP represents the data from broilers fed antibiotic growth promoters, and GOD-P and GOD-A represent the data from broilers fed GOD produced by *Aspergillus niger* and *Penicillium amagasakiense*, respectively

^2^SEM: Standard errors of the mean

### Changes in the Caecal microbiota related to supplementation with different types of GOD

The differential bacteria in the GOD-A vs. CON, GOD-P vs. CON, AGP vs. CON, GOD-A vs. AGP, GOD-P vs. AGP, and GOD-A vs. GOD-P comparisons were analysed by using the Mann-Whitney U test at the genus level (Fig. 3). For 21-day-old broilers (Fig. 3C), compared with the CON diet, supplementation with AGP significantly increased the abundance of *Devosia* and *Halomonas* but decreased the abundance of *Coprobacillus*, *Dorea*, *Lachnospira*, *Brenneria*, and *Faecalibacterium*; supplementation with GOD-P significantly increased the abundance of *Halomonas*, *Virgibacillus*, and *Faecalibacterium* but decreased the abundance of *Eggerthella* and *Brenneria*; and supplementation with GOD-A significantly increased the abundance of *Halomonas* and *Holdemania*. Compared with the AGP group, the GOD-P group had significantly increased abundances of *Faecalibacterium*, *Brenneria*, *Coprobacillus*, *Dorea*, and *Persicobacter* and decreased abundances of *Devosia*. Meanwhile, significantly increased abundances of *Lachnospira* and *Holdemania* were identified in the GOD-A group compared with the AGP group. Moreover, significantly decreased abundances of *Alkaliphilus* and *Virgibacillus* were identified in the GOD-A vs. GOD-P comparison.

For 42-day-old broilers (Fig. 3D), compared with the CON group, supplementation with AGP significantly increased the abundance of *Sutterella* and *Collinsella* but decreased the abundance of *Anaeroplasma*, *Candidatus Arthromitus*, *Dehalobacterium*, *Desulfotomaculum*, *Anaerofustis*, *Bacillus*, *Coprobacillus* and *cc*_*115*; supplementation with GOD-P significantly increased the abundance of *Lactobacillus* and *Anaerofilum* but decreased the abundance of *Persicobacter* and *Rikenella*; and supplementation with GOD-A significantly increased the abundance of Lactobacillus but decreased the abundance of *Anaeroplasma*, *Desulfotomaculum*, and *Rikenella*. Compared with the AGP group, the GOD-P group had significantly increased the abundances of *Candidatus Arthromitus*, *cc*_*115*, *Faecalibacterium*, *Anaerofilum*, *Dehalobacterium*, *Desulfotomaculum*, *Dorea*, *Holdemania*, and *Coprobacillus* and decreased abundances of *Moryella*, *Sutterella*, *Collinsella*, and *Bifidobacterium*. Meanwhile, significantly increased abundances of *cc*_*115*, *Faecalibacterium*, and *Lactobacillus* and significantly decreased abundances of *Collinsella* and *Sutterella* were identified in the GOD-A group compared with the AGP group. Moreover, significantly increased abundances of *Coprococcus* and *Sutterella* and decreased abundances of *Anaerofilum*, *Candidatus Arthromitus*, and *Desulfotomaculum* were identified in the GOD-A vs. GOD-P comparison.

### Changes in gut microbiota mediate the differential regulatory effects of the two different glucose oxidases and AGPs

**Fig. 4:**
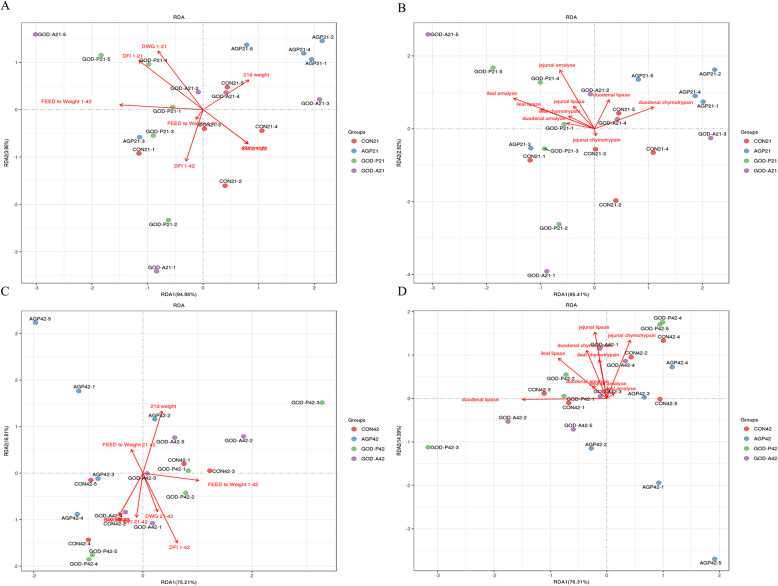
RDA analyses based on the identified differential genera of 21-day-old broilers (**a**-**b**) and 42-day-old broilers (**c**-**d**) revealed significantly altered growth performance and intestinal enzymatic activity-related indices. Note: CON indicates the control group, AGP represents the data from broilers fed antibiotic growth promoters, and GOD-P and GOD-A represent the data from broilers fed GOD produced by *Aspergillus niger* and *Penicillium amagasakiense,* respectively. For the relationship between groups and the identified altered phenotypes, if the plot projection of one sample occurred in the positive direction of the extending line of the identified altered phenotypes, then the treatment of this sample could promote this phenotypic change

RDA analyses were performed to identify the relationship among the identified differential genera of microbiota, broiler performance, and treatments (Fig. 4). As a result, we found that the AGP and GOD-P groups of 21-day-old broilers were all separately clustered in the area near improved growth performance and intestinal digestive enzymatic activities, while the CON and GOD-A groups were not well clustered, which indicated that supplementation with GOD and AGP was beneficial to microbiota fermentation, which was related to the increased growth performance and intestinal digestive enzymatic activities of 21-day-old broilers (Fig. 4A and B). However, the AGP group of 42-day-old broilers was clustered in the area near the increased ratio of feed intake to weight of 21–42 days and the decreased intestinal digestive enzyme activities. This results indicated that supplementation with AGP resulted in increased microbiota which is not beneficial to the growth of broilers from 21 to 42 days of age (Fig. 4C and D). Furthermore, according to the Spearman correlation analyses, several key genera that were separately correlated with growth performance, meat quality, antioxygenic properties, and intestinal enzymatic activities were also further identified and are listed in Table 3. Overall, the altered gut microbiota and the identified differential genera both suggested that the changes in the gut microbiota mediate the differential regulatory effects of the two different glucose oxidases and AGPs, especially the effects on the growth performance and intestinal enzyme activities of broilers. Specifically, the GOD-P group showed the most improved growth performance by regulating the gut microbiota; in brief, the significantly increased *Brenneria*, *Faecalibacterium*, and *Holdemania* were negatively related to the significantly decreased MDA content and were positively related to the significantly increased meat quality of 21-day-old broilers; the significantly increased *Brenneria*, *Virgibacillus*, and *Faecalibacterium* were positively related to the significantly increased intestinal enzyme activities of 21-day-old broilers; the significantly increased *Anaerofilum*, *Coprobacillus*, and *Dorea* were positively related to the significantly increased meat quality of 42-day-old broilers; and the significantly increased *Anaerofilum*, *cc_115*, and *Dehalobacterium* were positively related to the significantly increased intestinal enzyme activities of 42-day-old broilers.

**Table 3:** The key genera that were separately correlated with growth performance, meat quality, antioxygenic properties, and intestinal enzymatic activities by using Spearman correlation analyses

Genus	Performance	Correlation coefficient	*P-*value
21-day-old broilers			
*Alkaliphilus*	Ileal lipase (21-day-old)	0.656	0.002
*Brenneria*	pH_24h_ of breast muscle (42-day-old)	0.527	0.017
*Brenneria*	pH_24h_ of drumstick muscle (42-day-old)	0.570	0.009
*Brenneria*	Ratio of feed to gain (1–42 days)	0.475	0.034
*Coprobacillus*	pH_24h_ of breast muscle (42-day-old)	0.655	0.002
*Coprobacillus*	pH_24h_ of drumstick muscle (42-day-old)	0.480	0.032
*Devosia*	Duodenal lipase (21-day-old)	0.582	0.007
*Eggerthella*	Daily feed intake (1–21 days)	−0.537	0.015
*Faecalibacterium*	MDA (21-day-old)	−0.467	0.038
*Faecalibacterium*	Ileal amylase (21-day-old)	0.478	0.033
*Halomonas*	Daily feed intake (1–42 days)	−0.463	0.040
*Holdemania*	MDA (21-day-old)	−0.666	0.001
*Holdemania*	Daily weight gain (1–21 days)	0.470	0.036
*Holdemania*	Daily weight gain (1–42 days)	0.476	0.034
*Holdemania*	Body weight (42-day-old)	0.474	0.035
*Holdemania*	Jejunal amylase (21-day-old)	0.564	0.010
*Lachnospira*	Ratio of feed to gain (1–42 days)	0.474	0.035
*Virgibacillus*	pH_45min_ of drumstick muscle (42-day-old)	−0.575	0.008
*Virgibacillus*	Duodenal amylase (21-day-old)	0.576	0.008
*Virgibacillus*	Duodenal chymotrypsin (21-day-old)	0.464	0.039
*Virgibacillus*	Jejunal chymotrypsin (21-day-old)	0.451	0.046
*Virgibacillus*	Ileal lipase (21-day-old)	0.596	0.006
42-day-old broilers			
*Anaerofilum*	L* value of breast muscle	−0.497	0.026
*Anaerofilum*	L* value of drumstick muscle	−0.497	0.026
*Anaerofilum*	Ileal lipase (42-day-old)	0.612	0.004
*Anaerofustis*	Daily feed intake (1–42 days)	0.501	0.024
*Anaerofustis*	Duodenal lipase (42-day-old)	0.516	0.020
*Anaerofustis*	Ileal lipase (42-day-old)	0.562	0.010
*Anaeroplasma*	Daily feed intake (21–42 days)	0.460	0.041
*Anaeroplasma*	Duodenal chymotrypsin (42-day-old)	−0.553	0.011
*Bacillus*	Daily feed intake (1–42 days)	0.469	0.037
*Bifidobacterium*	L* value of breast muscle (42-day-old)	0.658	0.002
*Bifidobacterium*	pH_45min_ of drumstick muscle (42-day-old)	0.677	0.001
*Bifidobacterium*	pH_24h_ of drumstick muscle	0.597	0.005
*Bifidobacterium*	L* value of drumstick muscle (42-day-old)	0.658	0.002
*cc_115*	Duodenal lipase (42-day-old)	0.453	0.045
*Coprobacillus*	pH_45min_ of breast muscle (42-day-old)	0.461	0.041
*Dehalobacterium*	Ileal lipase (42-day-old)	0.516	0.020
*Desulfotomaculum*	MDA (42-day-old)	0.460	0.041
*Dorea*	L* value of breast muscle (42-day-old)	−0.512	0.021
*Dorea*	L* value of drumstick muscle (42-day-old)	−0.512	0.021
*Dorea*	Ratio of feed to gain (21–42 days)	−0.472	0.036
*Moryella*	L* value of breast muscle (42-day-old)	0.552	0.012
*Moryella*	L* value of drumstick muscle (42-day-old)	0.552	0.012
*Moryella*	Duodenal lipase (42-day-old)	−0.575	0.008
*Moryella*	Ileal lipase (42-day-old)	−0.533	0.016
*Persicobacter*	Body weight (21-day-old)	0.529	0.017
*Persicobacter*	Jejunal amylase (42-day-old)	−0.553	0.011
*Rikenella*	pH_24h_ of breast muscle (42-day-old)	0.476	0.034
*Rikenella*	Daily weight gain (1–42 days)	−0.479	0.033
*Rikenella*	Body weight (42-day-old)	−0.479	0.033
*Rikenella*	Ileal chymotrypsin (42-day-old)	−0.623	0.003
*Rikenella*	Ileal lipase (42-day-old)	−0.490	0.028
*Sutterella*	L* value of breast muscle (42-day-old)	0.509	0.022
*Sutterella*	L* value of drumstick muscle (42-day-old)	0.509	0.022

## Discussion

Two different GODs significantly improved the growth performance of broilers, which significantly promoted the ADFI and ADG of broilers during days 1–21. Many studies have shown that GOD could stimulate the growth response of broilers [4, 22]. According to previous studies, glucose oxidase catalyses the oxidation of β-*D*-glucose to gluconic acid by utilizing molecular oxygen as an electron acceptor with the simultaneous production of hydrogen peroxide [11]. The production of hydrogen peroxide could reduce intestinal pH and then further stimulate intestinal enzyme production and increase intestinal activities [23], which have been proven to stimulate the average intake and body weight of broilers. Hence, regarding the potential mechanisms of the 2 GODs in regulating the growth performance of broilers, the changed intestinal enzymatic activities in the two GOD groups suggested a potential improvement in the nutrient digestibility of broilers [24], which could potentially underlie the improved growth performance of the broilers in the two GOD groups.

The shear force, pH, drip loss, and colour are important indices for evaluating the meat quality of broilers. Similar to previous results [3, 4], the present study indicated that the pH_24 h_ and the L* values of the broilers were altered by supplementation with the 2 different GODs. It can be concluded that dietary supplementation with GOD, to some extent, improved the meat quality of broilers. The improvement of the meat colour index a might be related to the increased antioxidant capacity [25]. GOD consumes large amounts of oxygen in the gut and could therefore serve as an important antioxidant in animals by eliminating oxygen free radicals and then promote antioxidant capacity by upregulating hepatic and jejunal Nrf2/Keap1 [26], which could serve as the key causes of the improved meat quality of broilers. In addition to the increased nutrient digestibility induced by GOD supplementation, the improvement in antioxidant status was also considered to improve meat quality in the present study [27]. In a previous study, the metabolite MDA was associated with lipid peroxidation and avian stress [28]. In addition, MDA is widely used as an indicator of oxidative stress [29]. Supplementation with GOD had a beneficial effect on the antioxidative capacity and immunity of broilers [4]. The previous findings are consistent with our results that MDA content was decreased in both GOD treatments, indicating that GOD enhanced the antioxidant capacity of the broilers and then served as the main cause of the improved meat quality [3, 4].

The GODs produced by *A. niger* and *P. amagasakiense* could have differential roles in regulating the growth performance and meat quality of broilers. In view of that, the most significant improvement in growth performance and intestinal enzyme activities were identified in the GOD-P group, while all these indices were not as improved in the GOD-A group relative to the GOD-P group. Moreover, lightness (L*) could be increased with the increased oxidative stress of broilers under environmental stress [22], which indicated a significantly decreased meat quality [30, 31]. The decreased ∆pH and the increase in pH_24 h_ indicated significantly improved chicken meat quality [32]. Significantly decreased L values and pH_24 h_ values were identified in 21-day-old and 42-day-old broilers supplemented with GOD produced by *P. amagasakiense* when compared with those supplemented with GOD produced *A. niger*, which indicated that the GOD produced by *P. amagasakiense* is more beneficial to the meat quality of broilers. Thus, the different roles of 2 GODs in regulating the intestinal enzymes and antioxidant status of broilers attracted our further attention. According to previous studies, the structure, amino acid content, carbohydrate content, and enzyme kinetics of the GODs fermented by *A. niger* and *P. amagasakiense* were quite different [11]. These differences may underlie their different functional efficiencies in the regulation of the intestinal enzymatic activities and antioxidative function of broilers. Specifically, previous studies have speculated that GOD could influence the intestinal environment by utilizing oxygen, producing gluconic acid, and hydrogen peroxide, which are harmful to pathogenic bacteria and are beneficial to the survival of beneficial bacteria [4, 33]. Hence, the differential roles of GOD produced by *A. niger* and *P. amagasakiense* in regulating intestinal digestive enzymatic activities, antioxidant status, growth performance, and meat quality may be induced by the differential microbiota [34–36].

In the present study, supplementation with GODs from either *A. niger* or *P. amagasakiense* could significantly improve both the growth performance and intestinal enzymatic activities compared with the CON diet. Hence, the significantly increased *Halomonas* in 21-day-old broilers and *Lactobacillus* in 42-day-old broilers were co-increased in the two GOD groups compared with the CON group. *Halomonas* and *Lactobacillus* are probiotics in the gut, and supplementation with these 2 probiotics has been widely proven to be beneficial for promoting growth performance and increasing intestinal enzymatic activities [9, 37, 38]. Hence, these two bacteria could serve as key bacteria that play key roles in promoting growth performance and increasing intestinal enzymatic activities in the two GOD groups compared with the CON group. Furthermore, based on Spearman correlation analysis and RDA analysis, several differential genera in the GOD-P and GOD-A groups could contribute to the increases in growth performance and antioxidant ability. Specifically, of these genera, *Holdemania* [39]*, cc*_*115* [9], and *Dehalobacterium* [1, 40] were positively correlated with growth performance and antioxidant ability. Overall, the gut microbiota is reshaped by 2 different GOD supplements and further contribute to improve the antioxidant status and growth performance of broilers.

Furthermore, the differential genera in the GOD-P and GOD-A groups could also contribute to the differential roles of GOP-P and GOD-A in regulating meat quality and intestinal enzyme activities. According to a previous study, *Faecalibacterium* [41], *Virgibacillus* [42]*, Anaerofilum* [43], *Dorea* [44]*,* and *Coprobacillus* [35], which were significantly increased in the GOD-P group compared with the GOD-A group, have been suggested to increase the antioxidant ability and absorption of energy according to previous studies [35, 41–44]. Hence, the better regulatory roles of GOD-P might be attributed to its regulatory roles in changing the gut microbiota, which may be beneficial to the growth performance and meat quality of broilers.

## Conclusion

The gut microbiota is reshaped by 2 different GOD supplements produced by *A. niger* and *P. amagasakiense*; meanwhile, the altered gut microbiota could further influence the intestinal digestive enzymatic activities, antioxidant status, growth performance, and meat quality of broilers. In conclusion, the altered gut microbiota induced by supplementation with glucose oxidase produced by *P. amagasakiense* mediate better regulatory effects on the meat quality and growth performance of broilers than that induced by supplementation with glucose oxidase produced by *A. niger*.

## Data Availability

The sequence data were deposited and are available in the Sequence Read Archive (SRA) of NCBI with the accession project numbers PRJNA606870.

## Abbreviations

ADG: Average daily weight gain
ADFI: Average daily feed intake
AGPs: Antibiotic growth promoters
F/G: Ratio of feed to gain
GOD: Glucose oxidase
GSH-Px: Glutathione peroxidase
MDA: Malonaldehyde
OTUs: Operational taxonomic units
PCoA: Principal coordinated analysis
qRT-PCR: Quantitative real-time PCR
SOD: Superoxide dismutase
T-AOC: Total antioxidant capacity
